# Comparing Patient Perspectives on Diabetes Management to the Deficit-Based Literature in an Ethnic Minority Population: A Mixed-Methods Study

**DOI:** 10.3390/ijerph192214769

**Published:** 2022-11-10

**Authors:** Kathleen Abu-Saad, Nihaya Daoud, Giora Kaplan, Arnona Ziv, Arnon D. Cohen, Liraz Olmer, Daphna Pollack, Ofra Kalter-Leibovici

**Affiliations:** 1 Gertner Institute for Epidemiology and Health Policy Research, Sheba Medical Center, Ramat Gan 52621, Israel; 2 Department of Public Health, Faculty of Health Sciences, Ben-Gurion University of the Negev, Beer Sheva 84015, Israel; 3 Siaal Research Center for Family Medicine and Primary Care, Faculty of Health Sciences, Ben-Gurion University of the Negev, Beer Sheva 84015, Israel; 4 Department of Epidemiology and Preventive Medicine, School of Public Health, Sackler Faculty of Medicine, Tel-Aviv University, Tel-Aviv 6997801, Israel

**Keywords:** deficit discourse, racial/ethnic minority, diabetes, patient perspectives, mixed-methods, Arabs, Israel

## Abstract

Marginalized racial/ethnic minorities have disproportionately high rates of type 2 diabetes prevalence, complications and mortality. Researchers and policymakers have typically addressed these disparities using a deficit-based discourse focused on individual/cultural deficiencies or failure. A mixed-methods study was used to compare the deficit discourse to the perspectives of adults with diabetes in the Arab minority in Israel, using data from 10 focus groups (5 men’s, 5 women’s) and 296 quantitative in-person surveys. Both qualitative and quantitative data were triangulated. In addition, multivariable regression models tested associations between diabetes management perspectives and participant characteristics. Contrary to the deficit-based characterizations of patients as fatalistic and unknowledgeable, participants viewed diabetes as a chronic disease with serious complications. They expressed more support for patient responsibility in diabetes management than for passive fatalism, and were less fatalistic as educational level and adequacy of diabetes self-care training increased. The impact of social/environmental barriers and changing cultural norms on lifestyle behaviors was highlighted. Over 95% used prescription medications for diabetes management, although 35% reported economic barriers. The deficit discourse is not well-aligned with Arab patients’ evolving perceptions and needs, and has deflected attention from the socioeconomic/structural determinants of health, and the healthcare system’s responsibility to provide effective, culturally-relevant diabetes services.

## 1. Introduction

Marginalized racial/ethnic minorities have disproportionately high rates of type 2 diabetes prevalence, complications and mortality, as compared to their majority counterparts [1,2,3,4]. Healthcare researchers and policy makers have often responded to such disparities with a deficit-based discourse that focuses on individual and/or community deficiency, lack or failure (e.g., poor health literacy, poor health behaviors, non-compliance with treatment regimens, social dysfunction) [5,6]. This deficit discourse produces a static, monolithic definition of people that focuses practice and policy initiatives on fixing their assumedly inherent cultural or individual shortcomings (e.g., fatalism, ignorance). This framing implicitly or explicitly stigmatizes marginalized people/communities, and attributes the responsibility for their poor health outcomes to them alone, while overlooking the responsibility of healthcare providers and systems to supply effective, culturally appropriate services [5,6,7]. Deficit-based approaches also obscure the role of the social determinants of health (SDOH) and entrenched structural inequalities in shaping health outcomes, although socioeconomic and environmental factors have been found to account for 50–60% of health outcomes [5,8,9].

Over the past several decades, health researchers in Israel have produced a substantial body of literature on diabetes-related health disparities between the Arab minority and Jewish majority populations, which overwhelmingly exhibits a deficit-based approach [3,4,10,11,12,13,14,15,16]. The Indigenous Palestinian Arabs, who became citizens of Israel after 1948, have experienced a legacy of displacement, dispossession, and social, economic, geographic and political marginalization [17,18,19], similar to Indigenous/marginalized minorities in other parts of the world [20]. Currently, they comprise 20.9% of the total Israeli population [21]. They have a much higher poverty rate (50.3%) than the national average (21.2%), even after governmental transfer payments for the alleviation of poverty [22]. They are also highly segregated from the Jewish community residentially [18,23]; this, together with their high poverty rate, has a negative impact on health [17,18]. The age- and sex-adjusted prevalence of adult-onset diabetes is higher among Arabs (18.4%) than Jews (10.3%), as is the annual diabetes incidence rate (2.9% vs. 1.7%, respectively) [4]. Arabs with diabetes also exhibit lower rates of adequate glycemic control than their Jewish counterparts (33% vs. 53%, respectively) [13], and are generally younger when adult-onset diabetes is diagnosed [3,15,24]. The diabetic complication and cause-specific diabetes mortality rates are also higher among Arabs than Jews [23,24,25,26]. These disparities exist despite Israel’s national health insurance program, which provides all residents with community-based primary and regional secondary and tertiary healthcare services. Most Arabs, however live in peripheral regions of the country, and have suboptimal secondary- and tertiary-level service access [27].

Given the context of national health insurance in Israel, which ostensibly reduces healthcare access barriers, explanations for the disparity in the primarily deficit-based literature have turned to cultural or social factors, which are summarized in Box 1.

Box 1 **Common explanations for inadequately controlled diabetes among Arabs in Israel in the deficit-based literature.**

1. Inadequate understanding of the disease, its seriousness, and complications [11,13,16]
2. Fatalism [12,13]
3. Resistance to lifestyle change; traditional gender roles create barriers to women engaging in physical activity [10,12,13,15,16]
4. Distrust of modern medicine, low medication adherence, belief in traditional medicine [10,11,13,14,16]

In the current paper, we use data from an exploratory mixed-methods study to report minority patients’ perspectives on their diabetes management resources, challenges and needs. We then juxtapose their perspectives with the conclusions that have emerged from the deficits-based biomedical literature.

## 2. Materials and Methods

The Diabetes in the Arab population in Israel (DAPI) study was an exploratory, sequential mixed-methods study that employed focus groups to collect qualitative data, and an interviewer-administered survey to collect cross-sectional quantitative data from a sample of Arab adults with diabetes.

For the study sample, we selected five Arab communities that represent the geographic and socio-economic variation of the population (one city and village in the northern region, one city and mid-sized town in the central region, and one city in the southern region). We conducted the study in cooperation with Clalit Health Services (CHS), which is the healthcare organization that provides coverage to most of the Arab population in Israel (~70%) [28]. The study inclusion criteria were: (1) medical record diagnosis of diabetes, (2) aged 25–64 years, (3) non-institutionalized (community-dwelling) member of CHS in one of the study towns, (4) and physically and mentally capable of participating in the focus group or in-person survey interview. Exclusion criteria included: (1) erroneous diabetes diagnosis in the medical record, and (2) change of healthcare organization, town of residence, or community-dwelling status prior to first contact from study staff.

A list of study candidates was abstracted from the CHS electronic database, and included patients from the clinics of the study towns who had a diagnosis of diabetes. The abstracted data included patients’ place of residence, age, sex and contact information (n = 7847). In the qualitative study phase, a list of potential participants was randomly selected from the pool of patients by town (including 100 names per town). The study coordinator contacted candidates from the list sequentially and invited them to participate in a focus group. In the quantitative phase, a second list of candidates was randomly selected from the patient pool, excluding those who had been in the focus group participant pool. This list of candidates was also stratified by town (100 names per town), and provided to the Arab study interviewers, who contacted and recruited the participants sequentially. If candidates could not be traced, did not meet the eligibility criteria, or refused to participate, an alternative candidate was selected from the patient pool, who was matched according to age group, sex and town.

The sample size calculation was based on Sakar et al.’s [29] work, which indicated that a sample of 300 would provide 85% power to detect differences in diabetes self-management behaviors, by a one standard deviation increase in the self-efficacy score with an odds ratio of 1.46, and a two-tailed alpha of <0.05. We, thus, aimed to include 60 participants from each town in the quantitative sample. Of the quantitative survey candidates contacted (n = 345), 49 were not interviewed. Over half (n = 26) were not eligible due to physical or mental health problems, residence in a permanent care facility, erroneous classification as diabetic, death, or a change in healthcare organization or town of residence. The remaining 23 were eligible candidates who either refused to participate (n = 14) or could not be traced (n = 9). The final sample included 296 participants, which represented a response rate of 92.8% among eligible candidates. The in-person, interviewer-administered surveys were conducted from July 2012 to June 2013.

The study was conducted in accordance with the Declaration of Helsinki, and was approved by the Institutional Review Board of Sheba Medical Center. The participants in both phases of the study signed written, informed consent forms prior to their participation.

The qualitative data were collected via 10 focus group discussions (one for women and one for men in each study town) that were conducted in local CHS clinics. The average focus group included seven participants (range: 3–15). The participants filled in a short, anonymous demographic questionnaire prior to the focus group discussions, and consented to the audio-recording of the sessions. The focus groups were conducted in Arabic by trained Arab moderators, who began the discussion with a short vignette about a typical person with diabetes. They then posed open-ended questions about facilitators and barriers to this hypothetical person achieving optimal glycemic control (see Additional file S1 for the discussion guide). The focus group recordings were transcribed in the original language (Arabic).

For the quantitative data collection phase, we reviewed existing questionnaires related to various aspects of diabetes care together with the preliminary qualitative synthesis of the DAPI participants’ experiences of diabetes management. Given the exploratory nature of our study and our goal of centering patient perspectives, we used the focus group data to guide the process of selecting, adapting, and/or developing questions that best captured the issues of interest from the focus groups. We triangulated the qualitative and quantitative data, using the qualitative data to shape the questionnaire for the quantitative data collection, so that the quantitative results could shed light on the generalizability of issues and perspectives that emerged from the qualitative data synthesis. The questionnaire included information about socio-demographic parameters, medications taken for diabetes and all other chronic conditions (which participants were asked to provide at the in-person survey interview), general and diabetes-specific health status, self-management behaviors (e.g., diet, physical activity, self-blood glucose monitoring [SBGM]), healthcare service utilization, and adequacy of diabetes care provision (using selected questions from the Patient Assessment of Chronic Illness Care [PACIC] questionnaire [30]) and self-management education. It also included questions on the facilitators and benefits of, and barriers to, maintaining good glucose control. These questions were adapted from existing questionnaires (Health Belief Model Scale [31], ADA Diabetic Complications Risk Assessment Questionnaire [32]), or based on key statements/perspectives that emerged from the focus groups (for more details see Additional file S2). The questionnaire was piloted among 15 people with diabetes from a non-study Arab town. We used feedback from the pilot to modify several questions and improve their construct validity (e.g., ensure that questions measured the concepts they were intended to measure). The quantitative survey data were collected in face-to-face interviews either in a local CHS clinic (37.2%) or in participants’ homes (62.8%). Hemoglobin-A1c (HbA1c) test results were obtained from participants’ electronic health records.

The analysis of the qualitative data was based on a grounded theoretical inductive approach. Inductive approaches elicit information about people’s experiences and insights, and are grounded in the participants’ perspectives. The theory is, therefore, not taken from existing literature; rather, it is developed, based on empirical data, through the identification and prioritization of issues that are important to the study participants [33]. We followed a phenomenology methodologic approach [34] to develop a coding scheme through an iterative process, in which two team members (ND, KA) initially independently read/reviewed Arabic transcripts and audio-recordings and produced a list of topics. In addition, two of the transcripts were translated into Hebrew so that all core qualitative analysis team members (ND, KA, GK, OKL) could read them independently and identify possible themes. ND and KA consolidated the themes from all 10 focus groups, and reviewed them with the rest of the core analysis team in order to identify the key issues from the focus group discussions. These themes included factors used in the deficit-based literature to explain suboptimal diabetes management among Arabs (e.g., resistance to lifestyle/behavioral change, lack of understanding of the disease, fatalism, non-compliance with medication regimens), and are presented and further explored as the primary qualitative findings of the current analysis.

For the analysis of the quantitative data, we employed univariate analyses (using chi-square or Fischer’s exact tests for categorical variables, and t-tests or Wilcoxon tests for continuous variables) to explore participants’ socio-demographic characteristics, health-status, and healthcare and self-management experiences. A number of the deficit-based factors that were challenged in focus group discussions exhibited variability (>20%) in the quantitative data (e.g., fatalism, lack of understanding of disease severity, cultural barriers to outdoor exercise, use of traditional remedies), and we were interested in exploring the sources of variation. We, thus, examined the questionnaire statements representing these concepts as dependent variables in multivariable logistic regression analysis (dichotomized as Agree/Disagree), and tested potential explanatory variables (listed in Additional file S2) for significant and independent associations with the dependent variables. Co-variates were entered into models that controlled for sex (if relevant), age, and education, using the forward-stepwise method (with a *p*-value of <0.05 as the threshold for retention). SAS (version 9.4, SAS Institute Inc., Cary, NC, USA) was used to conduct all statistical analyses.

## 3. Results

The focus group participant characteristics are presented in Additional file S3-Supplemental Table S1. Most were in their mid-50s, married, and had median of 8 years of education. The participants were in their early to mid-40s when diagnosed with diabetes. Most (>90%) used oral hypoglycemic agents, while 44% of women and 63% of men used insulin.

The survey respondents were similar to focus group participants demographically, and exhibited high unemployment and low occupational status levels (Table 1). Their median diabetes duration was 9.9 years, and over half (52.4%) had HbA1c levels of 8.0% or higher. The majority (52.4%) were treated with insulin, either with or without oral hypoglycemic agents (OHA), while 45.3% were treated only with OHA. Nearly 80% had two or more chronic diseases in addition to diabetes, and 50.3% reported being disabled.

Family physicians were the diabetes caregivers for nearly 85% of respondents, while 13.2% received care from a diabetes specialist (Table 2). Over half of the participants had never visited a dietician (54.4%), and additional 33.1% had not visited a dietician in the previous year. Men rated the adequacy of the training they received for SBGM and complications prevention higher than women did. The respondents nearly universally rated medications (95.6%) as important to their glycemic control.

### 3.1. Understanding of Nature and Severity of Diabetes

The focus group participants spoke of diabetes as a very serious chronic disease that was dangerous because its effects did not manifest until it was too late to reverse them:

M4: Diabetes is a devious disease; you’re sick, but you don’t feel that you are sick. It slowly eats away at you, the way rust destroys iron, without you feeling it…like termites….

M5: Diabetes is a silent disease, you don’t know you have it…it’s not like a headache that you can feel…and by the time you start to feel its effects, it’s already too late.

**Figure 1:**
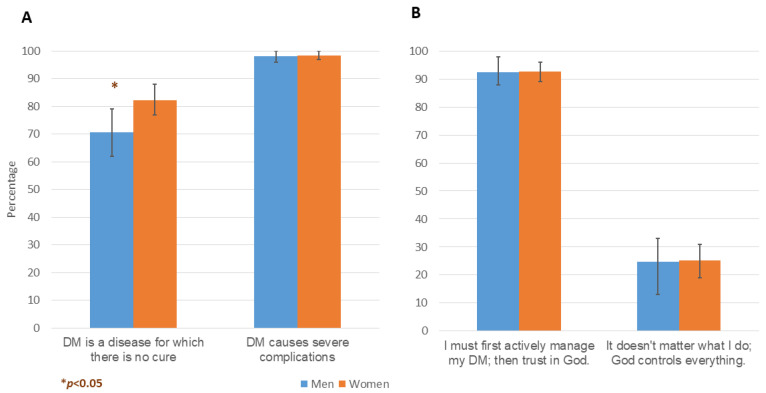
DAPI participant understanding of diabetes (Panel (**A**)) and attitudes toward its management (Panel (**B**)). The error bars represent 95% confidence intervals.

More female (82.3%) than male (70.6%) survey respondents viewed diabetes as an incurable disease (Figure 1A). Many of the focus group participants expressed a sense of discouragement and powerlessness in the face of this ‘silent disease’; while some viewed diabetes as a disease that could be successfully managed to avoid the complications:

W3: Diabetes never goes away. Maybe cancer is easier to deal with because it can be cured; but diabetes never goes away.

M2: Diabetes is an ‘elegant’ disease…if you keep eye on it, and don’t let your blood sugar get too high or too low, you can save yourself [from the complications]. It doesn’t kill you immediately. But if you don’t manage it, it can kill you very slowly.

In a multivariable logistic regression model controlling for age and sex, viewing diabetes as an incurable disease was positively associated with participants ever having consulted a diabetes specialist (OR: 2.42, CI: 1.17–5.00, *p* = 0.018), feeling factors beyond one’s control (e.g., economic/social/family/political) increased one’s blood sugar (OR: 2.12, CI: 1.10–4.10, *p* = 0.025), lacking help to conduct SBGM or interpret its results (OR: 2.53, CI: 1.24–5.13, *p* = 0.011), and not feeling full with the recommended diabetes diet (OR: 2.71, CI: 1.39–5.29, *p* = 0.004). In contrast, it was negatively associated with the educational level of participants (OR: 0.41, CI: 0.27–0.63, *p* < 0.001 per 1-level increase) (Additional file S3-Supplemental Table S3).

Nearly all survey respondents (98.3%) thought diabetes caused severe complications (Figure 1A). The focus group participants discussed their fear of diabetes complications, with some advising that fear be avoided because it would raise blood sugar levels. Others, however, considered the fear of complications a strong motivator for maintaining glycemic control:

M5-1: It’s better to die than to have your legs amputated and become disabled.

M5-2: Just don’t worry… don’t think ahead….

M3-1: I’m always afraid and this makes my blood sugar go up.

M3-2: If I’m afraid of developing complications, it makes me take better care of myself.

### 3.2. Fatalism

Fatalism (e.g., people absolving themselves of responsibility for managing their diabetes, and ‘leaving their fate to God’) arose in the focus groups, with participants discussing a complex range of responses to the question of who was responsible for controlling diabetes. As the following exchanges illustrate, fatalistic statements were often met with statements calling for active agency and responsibility to act, couched in religious and cultural maxims.

W5-1: [High blood sugar] is from God.

*Moderator: It’s from God? So we’re powerless to do anything? Whatever is destined to happen will happen?*

W5-2: No! What did God say-‘tie [up your camel] and then trust in God’.

The expression, “tie up your camel…” comes from the sayings of the Prophet Muhammad, who observed a man coming to the market, and leaving his camel free to roam. The Prophet advised him to secure his camel, but the man said there was no need because ‘he trusted in God.’ The Prophet responded, “Tie up your camel, and then trust in God.” This saying was repeated several times in the focus groups, with the application that people must first take an active part in their diabetes management, and then trust God to help them.

W3-1: We trust God.

W3-2: Take the medicine, and trust in God.

One woman, after describing her unsuccessful effort to avoid eating sweets at a wedding, concluded:

W1-1: Everything is in God’s hands. In a few years, we’re going to die anyway….

*Moderator: [When you eat like that], how high does your blood sugar get?*

W1-1: 300 to 350.

W1-2: That’s high, very high! Sister, you are not taking care of yourself.

W1-3: [Arabic saying:] “Three-grams of prevention is better than a third-ton of treatment.”

The influences of culture and religion were not by definition fatalistic, and often exerted the opposite influence. Most of the survey respondents (81.0%) defined themselves as religious, while fewer considered themselves either very religious (12.2%), or non-religious (6.8%) (Table 1). Nevertheless, 92.6% agreed with the statement, ‘According to the Prophet Muhammad, I must first “do my part”, and then trust in God; so I must take an active part in managing my diabetes’ (Figure 1B). Only 25.0% agreed with the statement, ‘It doesn’t matter what I do to manage my diabetes, in the end God controls everything’ (Figure 1B).

In multivariable analysis controlling for sex and age, a fatalistic approach to diabetes management was negatively associated with educational level (OR: 0.54, CI: 0.37–0.81, *p* = 0.003 per 1-level increase), higher self-rated adequacy of training for SBGM and complications prevention (OR: 0.52, CI: 0.29–0.92, *p* = 0.026), ever having visited a dietician (OR: 0.48, CI: 0.27–0.87, *p* = 0.015), and religiosity (religious vs. very religious: OR: 0.39, CI: 0.17–0.88; non-religious vs. very religious: OR: 0.12, CI: 0.02–0.66; *p* for type 3 analysis of effects = 0.020) (Additional file S3-Supplemental Table S4).

### 3.3. Resistance and Cultural Barriers to Lifestyle Change

Most survey respondents affirmed the importance of diet (91.6%) and physical activity (83.5%) to good diabetes management (Table 2). The focus group participants extensively discussed lifestyle behaviors, and the contextual factors that made it difficult for them to implement recommendations. The importance of individual motivation, willpower and self-control were often discussed. While many spoke of self-control as a challenge they struggled with and failed to achieve, others spoke of succeeding to exercise their willpower, and in turn succeeding to achieve glycemic control:

M3-1: I love sweets and fruit, and I can’t prevent myself from eating them while everyone around me is eating them.

M3-2: When you have to say no to everything, it’s just too hard.

M3-3: Everything goes back to the self-control and motivation of the person. If [a person with diabetes] wants to take care of himself, he will. If he doesn’t, no one can help him; not his wife or his children or the mother who gave birth to him.

W1: Our morale needs to be stronger than the disease.

Traditionally wheat was the main staple crop of this primarily agricultural population, and so bread was a major component of many meals [35]. Due to the population’s persistent poverty levels in Israeli society, bread remained important as an inexpensive food on which family members could ‘fill up’ [36,37]. This presented a particular dilemma for people with diabetes. Some participants discussed the need to eat healthier types of bread and described innovative initiatives they had taken to implement this, as well as barriers they faced to sustaining healthier behaviors.

W3: You have to eat the healthy bread the Jews make.

W4-1: I eat what we have. I don’t always have the things the dietician told me to eat…. The special diet bread is very expensive.

W4-2: I made bread with *inkhala* [wheat bran] and locally milled whole wheat flour instead of buying expensive whole wheat bread from [the Jewish market]...and my blood sugar levels went down. But then I got tired of doing this and went back to eating regularly, without any limitations, and my blood sugar went back up.

**Figure 2:**
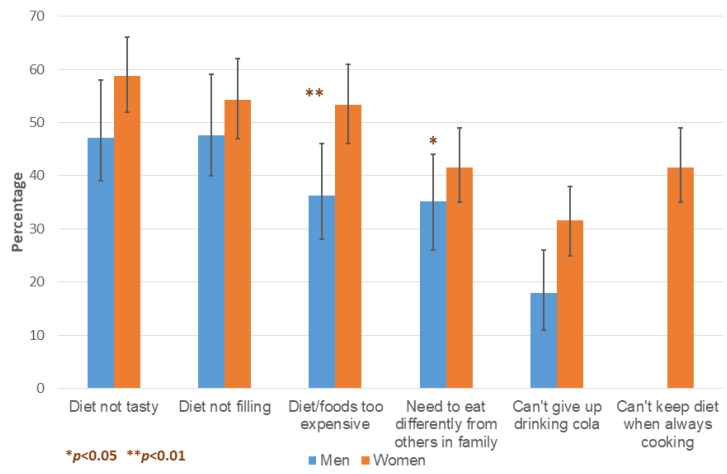
The reported barriers to implementing DM dietary recommendations. The error bars represent 95% confidence intervals.

The main barriers to implementing diet recommendations that emerged from the survey data are presented in Figure 2, and include individual, economic and social factors.

**Figure 3:**
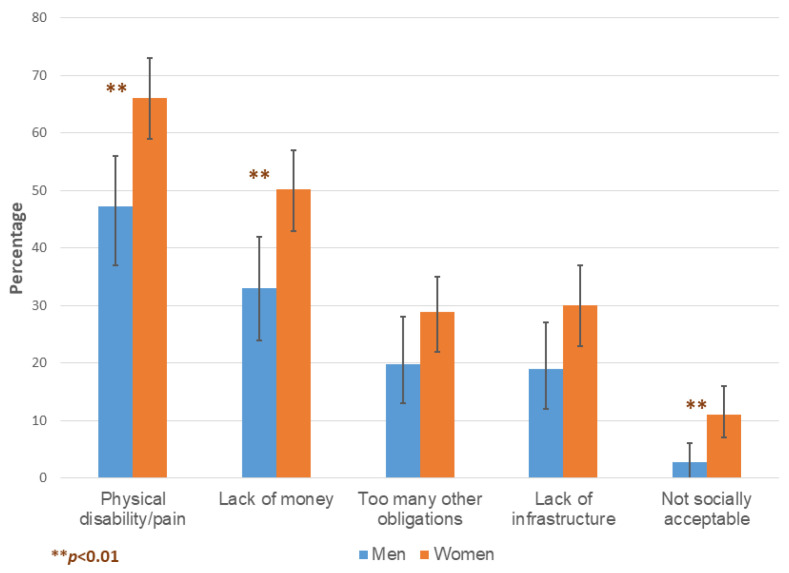
The reported barriers to implementing leisure physical activity (LPA) recommendations. The error bars represent 95% confidence intervals.

Physical disability/pain was the most frequently cited barrier to implementing leisure physical activity recommendations (Figure 3). Although cultural barriers to women engaging in outdoor physical activity have been highly stressed in the deficit-based literature, only 11.2% of female survey respondents indicated that this was a barrier for them. This issue was specifically probed by the moderator in the women’s focus groups, and their discussions indicated that these norms differed across communities/families and were continuously evolving.

*Moderator: How do you exercise?*

W5-1: I walk on the main street, and I walk fast.

W5-2: Yes, we see her and her husband out walking fast.

W5-3: I use a treadmill, 10 min a couple of times a day, because my husband doesn’t want me to walk in streets.

*Moderator: If you want to walk, where can you do it in your town?*

W2-1: Every place is full of cars [*a.n. many streets do not have sidewalks*]; it’s hard to find a place to walk.

W2-2: There is a nice walking area in [adjacent Jewish town], but it’s far away.

W2-3: There’s no place to walk here; the marketplace is too full of people.

*Moderator: Do you go out and walk in your town?*

W3: Yes, walking has become the fashion now.

The following exchange occurred in the women’s focus group of the most traditional community:

*Moderator: Where do you go to exercise?*

W4-1: We walk in the street.

*Moderator: Isn’t it a problem for you to walk there?*

W4-2: No, it’s not a problem.

W4-1: Walking is essential; it’s essential for every person to do it.

*Moderator: But not every woman can go out walking in the street!?*

W4-4: No, any woman who wants to can go out and walk, wherever she wants.

W4-1: [*Addressing the other focus group participants:*] She [the moderator] means that not every woman has permission from her husband go out walking in the streets.

*Moderator: Does that problem exist?*

W4-2: Yes, it does.

W4-5: Yes, but it’s very rare now.

W4-1: I used to have this problem, but my husband lets me go out walking now because the doctor came and visited us in our home and told him that he *has* to let me go out and walk, all the time [as part of my diabetes self-care].

*Moderator: Do you have any other solution? Do you have any place to exercise besides in the street?*

W4-2: No, we don’t have any other place; just on the streets.

In a multivariable logistic regression analysis including only the female survey respondents (n = 187), many potential factors were checked for associations with cultural barriers to outdoor exercise. In the final model, which was controlled for age and education, cultural barriers to engaging in outdoor exercise were positively associated with reporting that family/household responsibilities interfered with doing leisure physical activity (OR: 3.22, CI: 1.04–9.93, *p* = 0.042), and other social/family obligations disrupted their glycemic control (OR: 4.23, CI: 1.08–16.53, *p* = 0.038). In contrast, agreement with having the obligation to ‘act [to manage diabetes] and then trust in God’ was negatively associated with reporting cultural barriers to engaging in outdoor physical activity (OR: 0.03, CI: 0.01–0.16, *p* < 0.001) (Additional file S3-Supplemental Table S5). Education, marital status, religiosity, dislike of exercising, physical pain/mobility problems, economic stress and poor infrastructure were tested and were not found to be significantly and independently associated with cultural barriers to outdoor exercise in the multivariable model.

### 3.4. Non-Adherence to Prescribed Medications and Use of Traditional Medicines

Focus group participants spontaneously discussed traditional medicines/remedies and expressed a range of opinions about them:

M1: I drink tea with sage and ‘*zota*’, first thing every morning.

M3: I don’t believe in traditional medications; the scientists worked on the development of insulin for 20 years.

W2-1-Traditional remedies don’t work, they’re worthless; they don’t take the place of medicines.

W2-2-Lots of traditional remedies are sold in our town.

Although over 40% of the survey respondents reported a high perceived benefit of traditional remedies to glycemic control, this did not come at the expense of physician-prescribed medications, for which 95.6% of respondents reported a high perceived benefit (Table 2). Nearly all survey respondents reported using pharmaceutical agents for diabetes management (97.6%) and other chronic conditions (99.0%) (Table 1). In a multivariable model controlling for age, sex, and education, a high perceived efficacy of traditional remedies was positively associated with a high perceived efficacy for prayer/reading Quran (OR: 7.80, CI: 2.84–21.46, *p* < 0.001), and was negatively associated with having visited a dietician (OR: 0.48, CI: 0.29–0.80, *p* = 0.005), feeling highly susceptible to limb amputation (as a diabetic complication) (OR: 0.35, CI: 0.18–0.67, *p* = 0.006), and female sex (OR: 0.56, CI: 0.32–0.98, *p* = 0.042) (Additional file S3-Supplemental Table S6).

The focus group participants discussed other barriers to regularly taking physician-prescribed medications, most notably the costs of co-payments, particularly in families with limited incomes and multiple family members with chronic diseases:

M4: You do what you can, buy a little bit of food to eat, and a little bit of medicine, divided up, so you take a little of the diabetes medicine, and a few of the cholesterol pills, and a few of something else, and that’s how you keep yourself going with what you’ve got.

Over one-third (34.5%) of survey respondents reported lacking adequate money to buy medications during the previous year, while 55.6% of women and 35.8% of men reported that the lack of adequate money to buy medicines/supplies for diabetes self-management worsened their glycemic control (Additional file S3-Supplemental Table S2).

## 4. Discussion

In the mixed-methods DAPI study, we compared Arab patients’ perspectives on diabetes management to findings from the deficit-based literature. While there was variation in participant outlooks, the great majority understood diabetes to be an incurable disease with serious complications. The DAPI participants also expressed more support for patient responsibility in actively managing their diabetes, bolstered by religious and cultural maxims, than for passive fatalism. While recognizing the importance of lifestyle behaviors, they provided insights into contextual barriers to implementing recommendations, as well as examples of changing behaviors and cultural norms. The participants overwhelmingly valued and utilized prescription medications for diabetes management, with traditional remedies complementing, rather than replacing, pharmaceuticals. Non-adherence to medication regimens was more likely to occur due to economic barriers. These findings expose a dissonance between patient perspectives and the deficit-based literature, which has implications for practice and policy.

The deficit-based literature has led to ‘pathologizing’ marginalized communities (e.g., as ignorant, problematic, uncooperative) while drawing attention away from other factors, such as the healthcare system, that affect diabetes management [5]. The DAPI findings highlighted healthcare system shortcomings, successes and underutilized resources. For example, participants stressed medication co-payments as a barrier [35]. Many policymakers and providers have maintained that Israel’s national health insurance minimizes/eliminates economic access barriers, and that Arab patients choose not to use limited economic resources for medications because they do not consider diabetes a severe disease [13]. However, a study on healthcare expenditures in Israel found that Arabs spent significantly more on medication co-payments than Jews did, due to higher household levels of medical need, and that the regressive effect of this expenditure introduced inequities into the national health insurance system [38,39]. The DAPI findings also indicated that multi-disciplinary teams (e.g., diabetes specialists, dieticians, diabetes educators) improved patients’ understanding of the disease and reduced fatalism; however, most participants did not have access to multi-disciplinary provider teams [37]. The most common barriers participants mentioned regarding physical activity were pain/physical limitations, indicating a need for more effective pain management, and exercise alternatives and/or physical therapy support for people with disabilities. One story of a physician’s interaction with a patient’s family member, where they explained the centrality of physical activity to diabetes care, enabled the patient to overcome cultural barriers to women’s outdoor exercise and set a visible precedent for that community. These patient perspectives and experiences are critical to identifying where the healthcare system has failed and succeeded to supply adequate and culturally responsive services, and where further system interventions are needed.

The deficit-based diabetes literature has also had a myopic individual-level focus, while largely portraying Arab culture negatively [10,12,13,16] and overlooking the impact of collective obligations and communal resources on diabetes management and, potentially, prevention. Although this literature has focused on cultural barriers to women engaging in physical activity, largely to the exclusion of any other barriers [15], DAPI findings indicated that this ‘norm’ was neither static nor widely generalizable. The minority of women who reported a cultural barrier to outdoor exercise also tended to report competing family responsibilities and obligations interfering with their diabetes self-management. Similarly, a study among Hispanics in the U.S. reported that ‘*familism’* (e.g., strong connection to large family networks) could become a barrier when family needs came before individual needs [40]. Particularly for women, greater family obligations and responsibilities reduced the time available for engaging in physical activity and other healthy behaviors. Studies conducted in collectivist societies have suggested that in such contexts, family-/community-targeted behavioral interventions might be more effective than individual-targeted interventions [37,41,42,43,44]. Approaching healthy lifestyle behaviors as a social obligation (inclusive of all family members), rather than an individual obligation for a person with diabetes, may benefit from the key cultural role women play in managing the family (e.g., which could be extended to organizing family exercise groups). Such an approach has the potential to simultaneously improve an individual patient’s diabetes management, and to aid in preventing diabetes among first-degree family members who have an elevated risk [37,44].

The DAPI findings did not support the deficit-based conflation of fatalism, religion and Arab culture into a force that prevented patients from engaging in diabetes self-management [12,13]. Instead, most DAPI participants exhibited strong support for patient agency and proactive diabetes self-management, rooted in cultural-religious maxims. This is consistent with studies among other populations with high levels of religiosity that have found religion/spirituality to be important to patients’ lives and diabetes management, without replacing personal responsibility for self-care [42,45,46,47].

The deficit-based literature’s individual-level, biomedical focus has also led to overlooking the SDOH as vital venues for intervention. Several DAPI findings illuminate how the SDOH may either facilitate or prevent optimal diabetes management. Higher educational levels were associated with lower levels of fatalism, and lower odds of considering diabetes an incurable disease. While the later appears counter-intuitive and should be explored further in future research, it may indicate that those with more education felt empowered to control their diabetes and prevent its complications. DAPI participants identified inadequate economic resources and poor community infrastructure/walkability as substantial barriers to following diet, medication and physical activity recommendations. These findings are consistent with an extensive body of evidence indicating that minority-majority diabetes disparities cannot be ameliorated without treating the root causes and eliminating minority-majority disparities in the SDOH [8]. Despite this body of evidence, we did not find any studies focusing on the role of the SDOH in the literature on Arab-Jewish diabetes disparities in Israel. Likewise, a literature review on diabetes among Arabs in Israel identified no interventions targeting the SDOH [15]. However, a recent qualitative study among Arabs with diabetes in one region of Israel, which broke from the deficit-based approach and was centered around patient perspectives, emphasized the centrality of the SDOH to patient concerns [42]. The current and previous [37] DAPI study publications corroborate this finding and extend it by including a cross-national sample of Arab communities, and quantitatively confirming the generalizability of the qualitative findings in a larger, representative patient sample. The DAPI publications further explicitly raise the need for interventions in the SDOH to target the root causes of Arab-Jewish diabetes disparities in Israel.

The deficit-based literature on this and other marginalized populations have provided an evidence base that perpetuates static patient stereotypes among providers [6,13,15,48,49]. Since populations continuously evolve, it is important that research reflects this dynamic process. Research itself, however, can be insulated from evolving patient perspectives when it is dominated by provider/system perspectives. For example, in the formative phase of Levin-Zamir et al.’s [13] qualitative study, they used healthcare experts to identify Arab patients’ needs and barriers, rather than obtaining this information directly from patients. Furthermore, in their published findings, patient perspectives were not conveyed via direct quotes, but rather via the researchers’ summaries and interpretations.

The need for providers to stay abreast of their patients’ evolving perspectives is a global challenge, particularly in marginalized populations. Healthcare provision is structured into short, biomedically-focused appointments, which limit providers’ opportunities for in-depth listening and understanding of patients’ perspectives and experiences, and their evolution over time [48,49,50,51]. A study in the general U.S. population found discordance between family physicians’ perceptions of the health beliefs of their patients with diabetes, and patients’ actual health beliefs [52]. Such discordance was even stronger among stigmatized population groups [48,53]. Furthermore, the assumption that ethnic diabetes disparities can be explained by culture-based differences in diabetes beliefs has been challenged by studies reporting that differences in beliefs about the causes, consequences, and management of diabetes were associated primarily with socioeconomic factors, rather than with ethnicity/culture [54].

The DAPI study has a number of strengths and limitations. The limitations include its exploratory and cross-sectional design, which, though important for generating hypotheses, prohibit drawing conclusions about sequential or causal effects. We did not find relevant validated questionnaires addressing the dissonance between patient perspectives and the deficit-based literature, and, thus, many of the questions were newly developed. This is characteristic of exploratory research, which requires an openness to adapting research methods, and is intended to produce new avenues for further exploration, rather than conclusive results. Some of the comparisons, although found to be statistically significant, may be imprecise because of the limited sample size, and should be confirmed in larger study samples.

The strengths of this study include the use of a sequential, mixed-methods design, which enabled reflecting on the deficit-based literature through the lens of patient perspectives/experiences. This represents a novel, underutilized approach, which may open avenues for more effectively addressing the needs and improving the outcomes of minority patients with diabetes. Exploratory research typically uses either qualitative methods in small samples, or quantitative methods lacking in-depth data. In the DAPI study, however, the use of mixed methods and the triangulation of qualitative and quantitative data enabled us to evaluate the generalizability and external validity of the qualitative findings in a randomly selected, representative sample of Arab patients.

## 5. Conclusions

The DAPI study findings suggest that the deficit-based literature on Arabs with diabetes in Israel has not kept pace with the evolving perceptions and expressed needs of Arab patients, while deflecting attention from the role of the SDOH, and the responsibility of the healthcare system to provide effective, culturally-relevant diabetes management services. Patient-centric approaches are needed, not only in clinical settings, but also in research and policymaking contexts, in order to better understand and address the barriers and unmet needs that marginalized/minority patients with diabetes experience. Patient input can help healthcare providers, researchers and policymakers move beyond deficit-based approaches, to approaches that recognize, respect and utilize patients’ individual and communal strengths and resources, and that also address the socio-economic and structural determinants of health.

## Figures and Tables

**Table 1:** The selected characteristics and diabetes-related behaviors of DAPI interview respondents by gender (n = 296).

	Total (n = 296)		Women (n = 187)		Men (n = 109)		*p* ^a^
Demographics							
Age, *median (IQR)*	57.1	(50.6–63.1)	57.6	(50.8–63.7)	56.7	(50.1–62.9)	0.286
Marital status, n (%)							
Married	239	(80.7)	132	(70.6)	107	(98.2)	<0.001
Single/divorced	23	(7.8)	22	(11.8)	1	(0.9)	
Widowed	34	(11.5)	33	(17.6)	1	(0.9)	
Educational level, n (%)							<0.001
None	54	(18.3)	50	(26.7)	4	(3.7)	
Grades 1–8	126	(42.7)	81	(43.3)	45	(41.7)	
Grades 9–12	92	(31.2)	46	(24.6)	46	(42.6)	
Post-secondary	23	(7.8)	10	(5.4)	13	(12.0)	
Currently unemployed, n (%)	233	(78.7)	168	(89.8)	65	(59.6)	<0.001
Occupational status level ^b^ (range 1–9), *median (IQR)*	9	(7–9)	9	(9–9)	8	(6–9)	<0.001
Religiosity, n (%)							<0.001
Very religious	36	(12.2)	25	(13.4)	11	(10.1)	
Religious	239	(81.0)	158	(85.0)	81	(74.3)	
Non-religious	20	(6.8)	3	(1.6)	17	(15.6)	
**Health status**							
BMI, *median (IQR)*	32.0	(28.1–36.6)	32.5	(29.1–36.9)	30.9	(27.1–35.0)	0.010
Age at DM diagnosis, *median (IQR)*	45	(38–52)	46	(38–53)	44	(37–50)	0.154
DM duration, *median (IQR)*	9.9	(6.3–15.3)	9.7	(5.9–15.6)	10.8	(6.6–15.0)	0.493
DM therapy, n (%)							0.179
Diet alone	7	(2.4)	6	(3.2)	1	(0.9)	
Oral hypoglycemic agents alone	134	(45.3)	90	(48.1)	44	(40.4)	
Insulin with or without oral hypoglycemic agents	155	(52.4)	91	(48.7)	64	(58.7)	
Number of DM medications, *median (IQR)*	2	(1–2)	2	(1–3)	2	(1–2)	0.226
Total chronic medications, *median (IQR)*	6	(4–9)	6	(4–9)	6	(4–8)	0.654
HbA1c (%), *median (IQR)*	8.0	(7.0–8.9)	7.9	(6.9–8.7)	8.2	(7.3–9.4)	0.038
2 or more chronic conditions (in addition to DM), n (%)	235	(79.4)	146	(78.1)	89	(81.7)	0.436
Disability, n (%)	149	(50.3)	99	(52.9)	50	(45.9)	0.241
Self-rated health status as poor, n (%)	200	(67.6)	131	(70.1)	69	(63.3)	0.231
Cigarette smoking, n (%)							<0.001
Never	184	(66.7)	15	(83.3)	39	(38.2)	
Current smoker	52	(18.8)	18	(10.3)	34	(33.3)	
Past smoker	40	(14.5)	11	(6.3)	29	(28.4)	

DAPI Diabetes in the Arab population in Israel, DM diabetes mellitus, IQR interquartile range. ^a^ *p* for chi-square or Fisher’s exact test for categorical variables, and for Wilcoxon test for continuous variables. ^b^ The highest level of participant or spouse; highest score (9) = lowest occupational status.

**Table 2:** The healthcare and self-care parameters, and perceptions of their adequacy/value among DAPI interview respondents by gender (n = 296).

	Total (n = 296)		Women (n = 187)		Men (n = 109)		*p* ^a^
Healthcare Provision							
Primary physician providing DM care, n (%)							
Family physician	251	(84.8)	156	(83.4)	95	(87.2)	0.101
Diabetes specialist	39	(13.2)	29	(15.5)	10	(9.2)	
Other	6	(2.0)	2	(1.1)	4	(3.7)	
Three or more visits to doctor providing DM care in past year, n (%)	271	(91.6)	173	(92.5)	98	(89.9)	0.437
Dietician visits, n (%)							0.960
Never	161	(54.4)	101	(54.0)	60	(55.0)	
None in past year	98	(33.1)	63	(33.7)	35	(32.1)	
≥1 in past year	37	(12.5)	23	(12.3)	14	(12.8)	
Received adequate training for SBGM and complications prevention: n (%)							
a. Measuring blood sugar at home	232	(78.4)	137	(73.3)	95	(87.2)	0.013
b. About actions to take if blood sugar is too low	170	(57.4)	96	(51.3)	74	(67.9)	0.013
c. About actions to take if blood sugar is too high	153	(51.7)	85	(45.5)	68	(62.4)	0.006
d. About possible complications if blood sugar is not adequately controlled	180	(60.8)	102	(54.5)	78	(71.6)	0.013
e. About how to prevent complications	165	(55.7)	94	(50.3)	71	(65.1)	0.007
Adequacy of training score (sum of items a–e; range 0–5), *median (IQR)*	4	(1–5)	3	(1–5)	5	(2–5)	<0.001
**DM Management Behaviors and Beliefs**							
Meet leisure physical activity recommendation (≥2.5 h/wk), n (%)	36	(12.2)	23	(12.3)	13	(11.9)	0.925
High perceived benefit to glycemic control of: n (%)							
Regular follow-up visits to doctor/nurse	256	(87.2)	158	(84.5)	100	(91.7)	0.231
Regular follow-up visits to dietician	190	(64.2)	116	(62.0)	74	(67.9)	0.445
Regular blood tests at clinic	262	(88.5)	162	(86.6)	100	(91.7)	0.200
Taking prescribed medications	283	(95.6)	179	(95.7)	104	(95.4)	0.352
Taking traditional remedies/folk medicine	120	(40.5)	70	(37.4)	50	(45.9)	0.426

DAPI Diabetes in the Arab population in Israel, DM diabetes mellitus, SBGM self-blood glucose measurement. ^a^ *p* for chi-square or Fisher’s exact test for categorical variables, and for Wilcoxon test for continuous variables.

## Data Availability

The data presented in this study are available on request from the corresponding author. The data are not publicly available because the participants did not consent to this.

## Supplementary Materials

The following supporting information can be downloaded at: https://www.mdpi.com/article/10.3390/ijerph192214769/s1, Additional file S1: Focus Group Discussion Guides; Additional file S2: Methodological Appendix; Additional file S3: Supplemental Tables. References [30,31,32,55,56,57] are cited in the supplementary materials.
