# Supplementary material for: Comparing Patient Perspectives on Diabetes Management to the Deficit-Based Literature in an Ethnic Minority Population: A Mixed-Methods Study
Source: Int J Environ Res Public Health. 2022 Nov 10;19(22):14769. doi: 10.3390/ijerph192214769 (PMC9691122; doi:10.3390/ijerph192214769)
Supplement: Supplementary file 1 [file ijerph-19-14769-s001.zip › Additional file S2-methods appendix-r.pdf]

Comparing patient perspectives on diabetes management to the deficit-based literature in an ethnic minority population: A mixed-methods study

Additional file S2. Methodological Appendix

1. Questionnaire development

The DAPI questionnaire included a number of validated questionnaires that are not relevant to the analysis in this paper, including: the Spoken Knowledge in Low Literacy in Diabetes (SKILLD) questionnaire [55], the Diabetes Management Self-Efficacy Scale (DMSES) [56], and the Patient Health Questionnaire (PHQ-9) [57].

In this exploratory study, our aim was to elicit and prioritize the perspectives and experiences of Arabs with diabetes, and to quantitatively assess their generalizability and external validity in a representative sample of patients.

We found that the focus groups participants' diabetes management perspectives and experiences did not align with what has been written about them in the biomedical literature, which was dominated by healthcare system perspectives. In constructing the quantitative questionnaire to check the generalizability of these findings, we identified or adapted a limited number of questions from validated questionnaires, including: the Patient Assessment with Chronic Illness Care (PACIC) questionnaire (questions 12, 13) [30]; the American Diabetes Association Diabetes Complications Risk Assessment-Irish adaptation (question 7) [32]; and, the Health Belief Model Scale-Turkish version (questions 10, 13, 26, 32, 33, 36) [31].

On the whole, however, existing questionnaires validated in Western contexts (or adapted to/validated in other cultures with minor modifications that maintained a Western cultural and biomedical framework) did not address the points of dissonance that emerged between DAPI participant perspectives and the biomedical literature. As a result, we developed questions based on contrasting statements from focus group participants and the deficit-based literature that directly captured the points of difference. For example, for the issue of fatalism and diabetes management, we included one statement reflective of the deficit-based literature ("It doesn't matter what I do to manage my diabetes, in the end God controls everything."), and an opposing statement which drew on a traditional religious saying that emerged repeatedly in the focus groups ("According to the Prophet Muhammad I must first 'do my part', and then trust in God; so I must take an active part in managing my diabetes."). The Arab study team members examined the content validity of the questions before administration of the questionnaire, which was then piloted among 15 people with diabetes from a non-study Arab town. A number of questions were modified based on feedback from the pilot to improve the construct validity (e.g., ensure that questions measured the concepts they were intended to measure). (For references, see the article reference list.)

2. Definition of co-variates used in multivariable analyses

All covariates were collected in the in-person survey interviews.

**Co-variates**	**Definition**
Co-variates in all models	
Age	Years, continuous
Sex (except model 3)	Female/Male, binary
Educational level	Levels, ordinal<br>None<br>1-8 years<br>9-12 years<br>Post-secondary
Model 1: Outcome – DM is a disease for which there is no cure (Y/N)	
Ever had consultation with diabetes specialist	Yes/No, binary
Factors beyond one's control increase one's blood sugar	Yes/No, binary
Have no one to help do SBGM or interpret results	Yes/No, binary
Recommended diabetes diet not filling	Yes/No, binary
Model 2: Outcome – Fatalism: It doesn't matter what I do to manage my DM; in the end God controls everything (Y/N)	
Self-assessed adequacy of DM SBGM and complications prevention training score	Points, continuous
Religiosity	Very religious/ religious/non-religious, categorical
Model 3: Outcome (women only)– Outdoor exercise is unacceptable in my community (Y/N)	
Family/household responsibilities prevent doing leisure physical activity	Yes/No, binary
Other social/family obligations disrupt glycemic control	Yes/No, binary
Have obligation to 'act [to manage diabetes] and then trust in God'	Yes/No, binary
Model 4: Outcome – Traditional remedies/medicines can greatly improve glycemic control (Y/N)	
Ever had consultation with a dietician	Yes/No, binary
High perceived efficacy for prayer/reading Quran	Yes/No, binary
High perceived susceptibility to limb amputations	Yes/No, binary

DM diabetes mellitus; SBGM self-blood glucose measurement
