# Supplementary material for: Comparing Patient Perspectives on Diabetes Management to the Deficit-Based Literature in an Ethnic Minority Population: A Mixed-Methods Study
Source: Int J Environ Res Public Health. 2022 Nov 10;19(22):14769. doi: 10.3390/ijerph192214769 (PMC9691122; doi:10.3390/ijerph192214769)
Supplement: Supplementary file 1 [file ijerph-19-14769-s001.zip › Additional File S3-Suppl tables-r2-2.pdf]

Comparing patient perspectives on diabetes management to the deficit-based literature in an ethnic minority population: A mixed-methods study

Additional file S3: Tables

Supplemental Table S1. Selected characteristics of DAPI focus group participants by gender

	Women (n=36)		Men (n=27)	
Age (y), *median (IQ range)*	54	(49-59)	57	(50-63)
Married, N (%)	25	(69.4)	24	(88.9)
Years of education, *median (IQ range)*	8	(5-8)	8	(5-10)
Currently unemployed, *N (%)*	35	(97.2)	21	(77.8)
Age at diabetes diagnosis (y), *median (IQ range)*	45	(40-50)	44	(35-50)
Diabetes treatment, *N (%):*				
Oral hypoglycemic agents alone, *N (%)*	34	(94.4)	26	(96.3)
Insulin with or without oral hypoglycemic agents, *N (%)*	16	(44.4)	17	(63.0)

DAPI Diabetes in the Arab population in Israel

Supplemental Table S2. Perceptions of disease and management facilitators and barriers among DAPI interview respondents by gender

	Total (n=296)	Women (n=187)	Men (n=109)	P
**Patient perceptions of diabetes, *n (%)***				
DM is a disease for which there is no cure	232 (78.4)	155 (82.3)	77 (70.6)	0.014
DM causes severe complications	291 (98.3)	184 (98.4)	107 (98.2)	1.000
**Patient role in DM management, *n (%)***				
'A person is his own doctor', so I will choose which of the doctor's instructions to follow.	176 (59.7)	111 (59.7)	65 (59.6)	0.860
'A person is his own doctor', so therefore I am responsible for carrying out my diabetes treatment on a day-to-day basis.	264 (89.2)	164 (87.7)	100 (91.7)	0.513
Things beyond my control raise my blood sugar level (e.g., financial situation; family, social, or political problems)	166 (56.3)	113 (60.8)	53 (48.6)	0.011
It doesn't matter what I do to manage my diabetes, in the end God controls everything	74 (25.0)	47 (25.1)	27 (24.8)	0.262
According to the Prophet Muhammad I must first "do my part", and then trust in God; so I must take an active part in managing my diabetes.	274 (92.6)	173 (92.5)	101 (92.7)	0.742
**Patient actions that can improve glycemic control, *n (%)***				
Regular physical activity	247 (83.5)	158 (84.5)	89 (81.7)	0.112
Adherence to recommended diet	271 (91.6)	169 (90.4)	102 (93.6)	0.221
SBGM	252 (85.2)	152 (81.3)	100 (91.7)	0.051

	Total (n=296)	Women (n=187)	Men (n=109)	P
**Social obligations and support, *n (%)***				
Family tensions/pressures prevent maintaining adequate glycemic control	232 (78.6)	157 (84.4)	75 (68.8)	0.002
Family obligations (e.g., weddings, family gatherings) prevent maintaining adequate glycemic control	138 (46.6)	97 (51.9)	41 (37.6)	0.018
Have no one to help me do/interpret the results of SBGM	110 (37.3)	73 (39.0)	37 (34.3)	0.414
**Economic resources and barriers*, n (%)***				
Lack resources for doing sports/leisure physical activity	130 (43.9)	94 (50.3)	36 (33.0)	0.004
Lack resources for buying recommended foods for DM management	140 (47.5)	100 (53.5)	40 (37.0)	0.007
Prevented sometimes/often in past year by economic situation from:				
Buying medications	102 (34.5)	67 (35.8)	35 (32.1)	0.516
Buying supplies for DM care (e.g., SBGM test strips, lances)	103 (34.8)	66 (35.3)	37 (33.9)	0.814
Making visits to the dietician	106 (35.8)	70 (37.4)	36 (33.0)	0.446
Making co-payments for specialist visits/services	69 (36.8)	49 (26.2)	20 (18.3)	0.123
Paying travel expenses for various clinic visits	109 (36.8)	73 (39.0)	36 (33.0)	0.301
Paying to visit private physicians	148 (50.0)	102 (54.5)	46 (42.2)	0.041
Obtaining dental care	150 (50.7)	106 (56.7)	44 (40.4)	0.007
Lack of money for buying medicines/supplies for DM self-management worsens my glycemic control	143 (48.3)	104 (55.6)	39 (35.8)	0.001

	Total (n=296)	Women (n=187)	Men (n=109)	P
Economic distress/ request help for:				
Purchasing medicines/supplies for DM self-management	178 (60.1)	126 (67.4)	52 (47.7)	0.001
Any aspect of DM care (e.g., medications, health/dental care and associated travel costs, diet, PA)	230 (77.7)	158 (84.5)	72 (66.1)	<0.001

DAPI Diabetes in the Arab population in Israel, SBGM self blood glucose measurement, DM diabetes mellitus, PA physical activity

Supplemental Table S3. Multivariable logistic regression model of factors associated with viewing diabetes as an incurable disease among DAPI interview respondents (n=296)

Factor	OR	95% CI		P
Age (per 1-y increment)	0.97	0.94	1.01	0.139
Female vs male	1.12	0.57	2.18	0.747
Educational level (per increase of 1 level)	0.41	0.27	0.63	<0.001
Ever had consultation with diabetes specialist: yes vs no	2.42	1.17	5.00	0.018
Factors beyond one's control increase one's blood sugar: yes vs no	2.12	1.10	4.10	0.025
Have no one to help do SBGM or interpret results: yes vs no	2.53	1.24	5.13	0.011
Recommended diabetes diet not filling: yes vs no	2.71	1.39	5.29	0.004

DAPI Diabetes in the Arab population in Israel, SBGM self-blood glucose measurement

C-statistic: 0.81

Supplemental Table S4. Multivariable logistic regression model of factors associated with a fatalistic approach to diabetes management among DAPI interview respondents (n=296)

**Factor**	**OR**	**95% CL**		**P**
Age (per 1-y increment)	0.99	0.96	1.02	0.525
Female vs male	0.55	0.29	1.05	0.072
Educational level (per increase of 1 level)	0.54	0.37	0.81	0.003
High self-assessed adequacy of diabetes SBGM and complications prevention training score: ≥4 vs <4	0.52	0.29	0.92	0.026
Ever had consultation with a dietician: yes vs no	0.48	0.27	0.87	0.015
Religiosity				0.020
Religious vs very religious	0.39	0.17	0.88	
Non-religious vs very religious	0.12	0.02	0.66	

DAPI Diabetes in the Arab population in Israel, SBGM Self-blood glucose monitoring

C-statistic: 0.70

Supplemental Table S5. Multivariable logistic regression model of factors associated with reporting cultural barriers to engaging in outdoor exercise among female DAPI interview respondents (n=187)

Factor	OR	95% CL		P
Age (per 1-y increment)	1.03	0.97	1.11	0.336
Educational level (per 1-level increase)	1.01	0.11	1.98	0.984
Family/household responsibilities prevent doing leisure physical activity	3.22	1.04	9.93	0.042
Other social/family obligations disrupt glycemic control	4.23	1.08	16.53	0.038
Have obligation to 'act [to manage diabetes] and then trust in God'	0.03	0.01	0.16	<0.001

DAPI Diabetes in the Arab population in Israel

C-statistic: 0.84

Supplemental Table S6. Multivariable logistic regression model of factors associated with attributing a high perceived efficacy of traditional remedies among DAPI interview respondents (n=296)

Factor	OR	95% CL		P
Age (per 1-y increment)	1.02	0.99	1.05	0.159
Female vs male	0.56	0.32	0.98	0.042
Educational level (per 1-level increase)	1.01	0.72	1.42	0.944
Ever had consultation with a dietician: yes vs no	0.48	0.29	0.80	0.005
High perceived efficacy for prayer/reading Quran: yes vs no	7.80	2.84	21.46	<0.001
High perceived susceptibility to limb amputations: yes vs no	0.35	0.18	0.67	0.002

DAPI Diabetes in the Arab population in Israel

C-statistic: 0.71
